# Image-Guided Prostate Cryoablation: State-of-the-Art

**DOI:** 10.3390/medicina59091589

**Published:** 2023-09-02

**Authors:** Vijay Ramalingam, Colin J. McCarthy, Spencer Degerstedt, Muneeb Ahmed

**Affiliations:** Beth Israel Deaconess Medical Center, Division of Vascular and Interventional Radiology, Harvard Medical School, Deaconess Rd, Rosenburg 3, Boston, MA 02215, USA; cmccar11@bidmc.harvard.edu (C.J.M.); sdegerst@bidmc.harvard.edu (S.D.); mahmed@bidmc.harvard.edu (M.A.)

**Keywords:** prostate focal therapy, prostate cryoablation, men’s health

## Abstract

Image-guided focal therapy has increased in popularity as a treatment option for patients with primary and locally recurrent prostate cancer. This review will cover the basic indications, evaluation, treatment algorithm, and follow-up for patients undergoing image-guided ablation of the prostate. Additionally, this paper will serve as an overview of some technical approaches to cases so that physicians can familiarize themselves with working in this space. While the focus of this paper is prostate cryoablation, readers will obtain a basic literature overview of some of the additional available image-guided treatment modalities for focal prostate therapy.

## 1. Introduction

### 1.1. Background and Diagnosis

Prostate cancer is a serious cause of morbidity and mortality with varying treatment algorithms worldwide. It is the second most common cancer in men. The worldwide prostate cancer burden will be approximately 2.3 million new cases with 740,000 deaths by 2040 due to an aging population and heterogeneity in screening protocols [1]. The majority of men with prostate cancer are diagnosed with localized disease and are increasingly being diagnosed at a younger age due to improved diagnostic capabilities and widespread screening protocols [2,3]. The current treatment algorithms vary from active surveillance to radical treatment strategies with or without hormonal therapies, all of which are associated with potential side effects [4,5,6,7]. Since the initial reports of prostate cryoablation nearly three decades ago, there has been interest in minimally invasive treatment options for patients with prostate cancer [8,9]. In the past decade, there has been an increase in the number of published reports of operators using minimally invasive therapies to treat prostate cancer utilizing several different energy sources and guidance modalities [4,10,11,12]. While a comprehensive review of the varying methods of targeted or focal therapy for prostate cancer is beyond the scope of this review, this topic was covered in multiple prior reviews [13,14]. Of note, all the current focal therapy modalities are limited by a lack of long-term data, as well as a lack of standardized selection criteria and post-procedure follow-up [7,13,15]. The purpose of this article is to review the current rationale and basic technique of image-guided prostate cryoablation. Herein, we discuss the relevant fundamental concepts to understand where prostate cancer cryoablation fits within the overall realm of prostate cancer therapy.

### 1.2. Prostate Cancer: An Overview

Within the field of prostate cancer treatment, interventional radiologists should be familiar with basic patient evaluation, biopsy options, and primary prostate cancer, as well as recurrent prostate cancer, given the differences in treatment strategies [11]. Historically, for all men with suspected prostate cancer, the evaluation began with a digital rectal exam and prostate specific antigen (PSA) screening, together with a physical exam and possible prostate biopsy [11,16]. In recent years, with the advances in cross-sectional prostate imaging, patients also commonly undergo multiphasic MRI (mpMRI) imaging and possibly additional fusion or targeted image-guided biopsies as part of the diagnostic work-up and treatment selection [17]. High-quality mpMRI studies provide valuable anatomic definition of the prostate and surrounding neurovascular and organ structures, delineation of the tumor, as well as pre-procedure planning for planned focal ablative or radical therapies [18]. As mpMRI imaging strategies have improved, there is increasing evidence that the addition of mpMRI to both biopsy algorithms and patient work-up improves the likelihood of diagnosing clinically significant cancer [19,20]. 

### 1.3. Primary Prostate Cancer

Once diagnosed with prostate cancer, the standard treatment algorithm for men with localized disease varies on a combination of risk stratification, Gleason score, and biopsy results [21,22]. Based on the results of several large studies in recent years demonstrating a large number of men being overtreated with radical treatments for prostate cancer, many studies now suggest active surveillance as a first-line option for low-risk prostate cancer, as well as favorable intermediate-risk prostate cancer. This strategy does carry the risk of the development of metastatic disease and is associated with frequent blood tests, patient follow-up, biopsies, imaging, and patient anxiety [3,11,23,24]. For patients with organ-confined intermediate-risk or high-risk disease who are counseled to be treated, the current recommendations are radical treatment with either surgical prostatectomy or whole gland radiation [21,25]. While both are associated with specific risks and benefits, surgical whole gland therapy was found to have a lower rate of cancer-specific mortality and disease recurrence [5,11,26,27]. Regardless of strategy use, all radical prostate cancer treatment strategies are associated with risks of urinary incontinence, erectile dysfunction, and negative quality of life impacts, which are of increasing concern to patients [27]. While recent advances in our understanding of prostate cancer have resulted in a greater use of initial surveillance over treatment, many patients eventually undergo treatment with current first-line therapies, such as surgical resection, external beam radiation, or brachytherapy, with or without androgen deprivation therapy (ADT), which are all associated with compromised quality of life [4,5,6].

### 1.4. Recurrent Prostate Cancer

Most patients who eventually undergo treatment for prostate cancer receive first-line radical therapy with either radiation prostatectomy or radical prostatectomy, with or without androgen deprivation therapy (ADT) [4,5,6]. However, almost 20–40% of these patients will develop local recurrence within 5–10 years after initial treatment [28]. While there are varying reports regarding the impact of untreated tumor recurrence, some studies have reported up to 18% mortality at 10 years [29].

Treatment options for locally recurrent prostate cancer remain limited, with the largest gap in prostate cancer treatment currently being the lack of available treatment options for those patients who recur after radical therapy. Repeat local radiation therapy is dose-limited, with a higher rate of radiation toxicity in the salvage setting such as bladder neck and urethral strictures, urinary incontinence, and poor tissue healing. Additionally, repeat radiation therapy is associated with a higher risk of recurrent disease [30,31,32]. Repeat surgical resection is also not suitable in all patients and is associated with significant side effects [33]. While the role of ADT is well established for metastatic disease, there are limited data supporting its use in biochemical or local recurrence [34,35]. ADT itself is associated with side effects, including thromboembolic events, fatigue, sexual and urinary dysfunction, and skeletal fractures, as well as the development of higher grade castration-resistant prostate cancer after years of treatment [28,36,37,38,39]. To that end, oligometastatic-directed therapy was studied as a method to delay the need for ADT [13]. Therefore, there is ongoing need for focal treatments that are locally effective.

## 2. Image-Guided Tumor Ablation in Prostate Cancer

Focal therapy with image-guided ablative modalities is a potentially attractive option for the treatment of focal lesions within the gland. The concept behind the use of focal therapies such as tumor ablation in this patient cohort is that by targeting only the cancerous region of the prostate gland, men could have their disease controlled while minimizing the risks of urinary and sexual side effects. Oncologically, the rationale behind targeted ablation therapy to the prostate lies in the idea that a single precursor cell is responsible for metastatic prostate disease [40]. Furthermore, this idea expands to explain the idea that while most prostate cancer is multi-focal within the gland, with refined biopsy and imaging techniques, the dominant area of disease (index lesion) can be identified and treated while monitoring the remaining gland with active surveillance, essentially down-staging a patient from high/intermediate risk disease to low-risk disease [15,40,41].

## 3. The Role of Cryoablation in Prostate Cancer

Based on the previously discussed issues with radical treatments for prostate cancer, as well as the potential challenges with existing options for recurrent disease, there is a need for image-guided ablation in this group of patients. Any discussion of focal therapy for prostate cancer requires an understanding of the broad definition of the image-guided ablation currently being described in the literature.

The initial reports of cryoablation presented were performed in the operating room with whole gland cryoablation. While these ablations were performed under imaging guidance, they were carried out by utilizing a standardized template with the goal of radical therapy while monitoring with transrectal ultrasound or temperature probes [8,9]. While ablation approaches have improved in recent years from whole gland approaches to more hemi-gland and focal approaches, the majority of the published image-guided approaches utilize ultrasound guidance in the operating room. In other cases, MRI/US fusion is employed, but needle or probe placement and thermal monitoring are performed with the use of ultrasound guidance [25,42].

To that end, interventional radiologists offer a unique ability to offer image-guided cryoablation utilizing a combination of ultrasound with advanced cross-sectional imaging where needle placement, as well as thermal monitoring of the cryoablation zone, can be performed. Early reports of cryoablation utilizing true MRI guidance were reported in 2014 when Gangi et al. reported their experience of 11 patients undergoing whole gland cryoablation with one reported major complication (urethro-rectal fistula). This was further replicated in 2018 when Kinsman et al. reported their initial experience of four patients who had whole gland MRI-guided prostate cryoablation with two patients reporting minor urinary symptoms, which resolved [43,44]. More recently, a 2019 study by De Marini et al. outlined results of 30 patients undergoing whole gland MRI-guided cryoablation with acceptable oncologic outcomes and no patients dying from prostate cancer. Notably, there was a 60 percent reported patient complication rate. Of the 18 patients with complications, 5/18 required surgical/interventional treatments, and the remaining 13 were managed conservatively or with pharmacological treatment. (This study was limited by a heterogeneous population, including both primary and salvage prostate cancer patients [45].

In the recurrent setting, Woodrum et al. published an initial series of 18 patients treated with MRI-guided focal cryoablation and demonstrated technical efficacy and safety and also demonstrated that a more sustainable treatment effect was achieved with a more aggressive ablation margin. Of note, there were no rectal or bladder complications, highlighting the benefit of real time isotherm monitoring with cross-sectional imaging [32]. This was further replicated in a study by Overduin et al., where focal cryoablation of recurrent disease was performed in 47 patients, and the risk of recurrence was correlated with an insufficient margin of the ice ball and a less aggressive protocol for ablation [46]. Similar results were reported in a study of nine patients in a paper by Bomers et al. [47].

### 3.1. Patient Selection for Cryoablation 

Patient selection for cryoablation remains an active area of interest. The 2022 AUA guidelines endorse whole gland or focal cryoablation as an option in intermediate-risk prostate cancer; however, it should only be recommended after other radical treatments such as radiation therapy or surgical prostatectomy with the disclaimer that there have been no head-to-head trials [48]. The AUA guidelines further recommend that patients with unfavorable intermediate-risk and high-risk prostate cancer only undergo ablation in the trial setting. However, in patients unwilling or unable to undergo standard treatments with intermediate-risk disease, cryoablation can be offered. Low risk patients who have engaged in shared decision-making may also undergo prostate cryoablation but should be counseled that, to date, there is little evidence supporting any substantial treatment benefit of ablation when compared to active surveillance.

Another area of active interest is the utilization of ablation for patients with prostate cancer after radical treatment failure or recurrence. Several trials have demonstrated local tumor control and the potential to defer the need for ADT or further radiation therapy [32,49]. While there are still many questions to be answered regarding appropriate patient selection, there is increasing interest in this area, and as a result, international consensus panels have been formed to evaluate this evolving area of treatment [50,51].

Ultimately, the decision to proceed with focal treatment for primary cancer relies on being able to adequately select low-risk/favorable intermediate-risk lesions where the index lesion is the highest-grade cancer and imaging demonstrates the index lesion with biopsy confirmation, unilateral disease, and finally, the ability to accurately target and monitor the ablation zone to treat the lesion while minimizing surrounding collateral damage [11,51]. The goal in image-guided ablation is to target the lesion with an adequate margin whilst also preserving the surrounding structures. In recent years, targeted therapy has been proposed as a superior option due to the increased risk of urinary and sexual side effects associated with whole gland and large volume ablation [32,46,52].

The management of patients with recurrent prostate cancer is based on careful discussion between the patient, oncologist, interventional radiologist, and other specialists to discuss the risks and benefits of treatment, the likelihood of disease progression, and the option of surveillance, coupled with the patient’s medical comorbidities and life expectancy. For well-selected patients with evidence of disease recurrence, it is important to rule out distant metastatic disease, as well as confirm localized recurrence, which is targetable by ablation. Generally, a rapid rise in PSA as well as high-grade disease are poor prognostic indicators [11,53].

The potential criteria for image-guided focal ablation of recurrent prostate cancer rely on biopsy-proven local recurrent disease that can be visualized by imaging with no distant metastases, confirmed by cross sectional imaging (CT, MRI) and/or advanced imaging such as PSMA PET-CT, as well as thorough patient counseling due to a higher risk profile in the salvage setting [11,49].

### 3.2. Technical Component 

It is important to note that there are no technical guidelines or accepted image guidance standards for prostate ablation. Institutional expertise with both ablation and imaging modalities are important determinants in how these patients are treated [54]. At our institution, all patients are evaluated in a multidisciplinary tumor board. Those selected for potential targeted ablation undergo additional evaluation by an attending interventional radiologist before the procedure. A dedicated history, physical examination, and urinary and sexual function scores are assessed in a dedicated clinic. All procedures are performed in the outpatient setting and followed up post-procedure in the interventional radiology clinic by the treating provider.

Procedures are commonly performed by two radiologists due to the technical complexity of the procedure. Given the lack of MRI guidance platforms readily available, all procedures are performed with CT and ultrasound guidance under general anesthesia. Following anesthesia induction, a urethral warming catheter (Boston Scientific, Inc., Marlborough, MA, USA) is placed over a 0.035-inch Glide Advantage Wire (Terumo Medical Systems, Shibuya City, Tokyo, Japan) to protect the urethra from thermal injury. The urethral warming catheter and guidewire are left in place throughout the treatment and for 15 min after the procedure and continuously circulate saline at 40 °C. 

**Figure 1:**
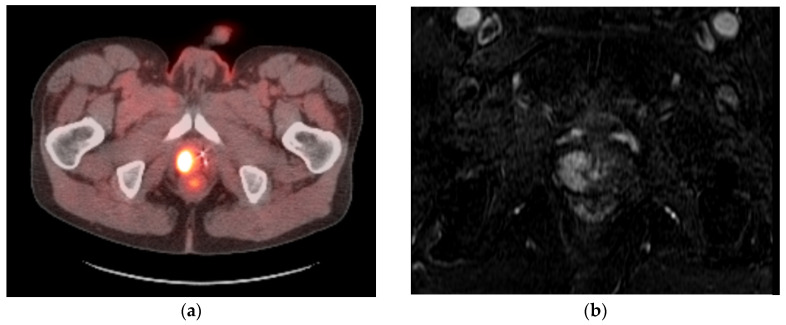

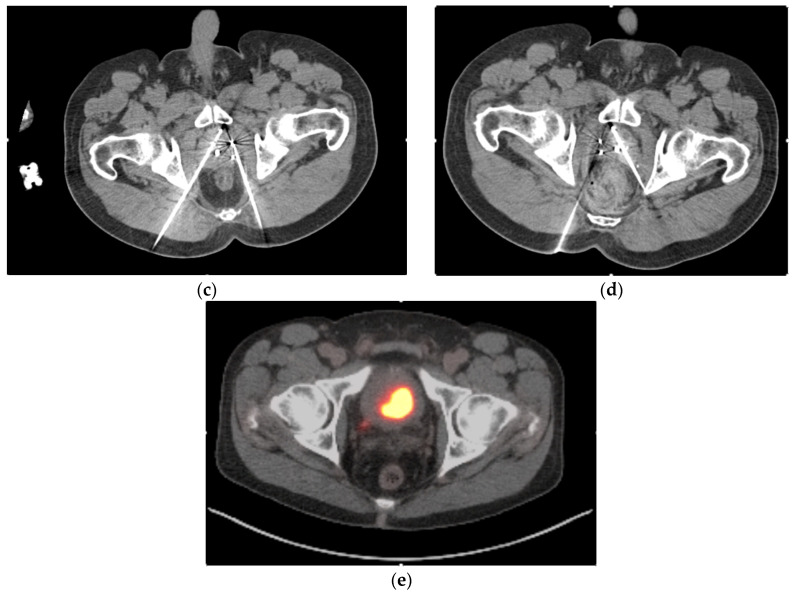
Case example of focal CT-guided cryoablation of locally recurrent prostate cancer. (**a**) Fused axial images of a fluciclovine-flourine-18-labeled SPECT/CT showing a focus of avidity along the right peripheral zone apex, correlating with local prostate cancer recurrence. (**b**) Axial contrast-enhanced T1-weighted MRI subtraction image showing the enhancement of locally recurrent prostate cancer within the right peripheral zone apex correlating with area of avidity (**a**). (**c**) Axial CT images showing transgluteal IcePearl cryoablation needle within the target lesion within the right hemi-prostate and placement of perirectal hydrodissection needle. (**d**) Transgluteal cryoablation probe into the prostate target lesion after instillation of 2% contrast:0.9% saline solution within the perirectal region to safely separate critical structures for cryoablation. (**e**) Fused axial images of the post-ablation 68Ga-PSMA-11 PET-CT showing no residual avidity along the right peripheral zone apex.

**Figure 2:**
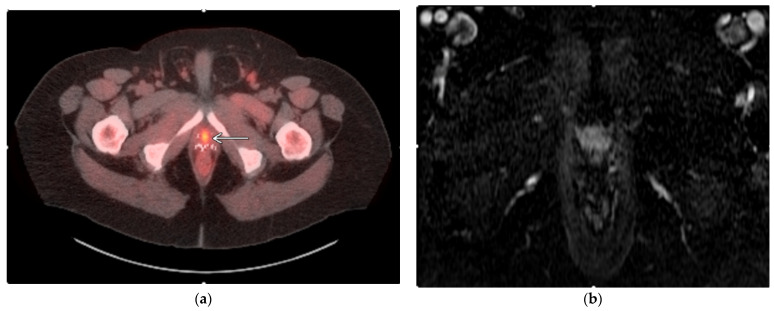

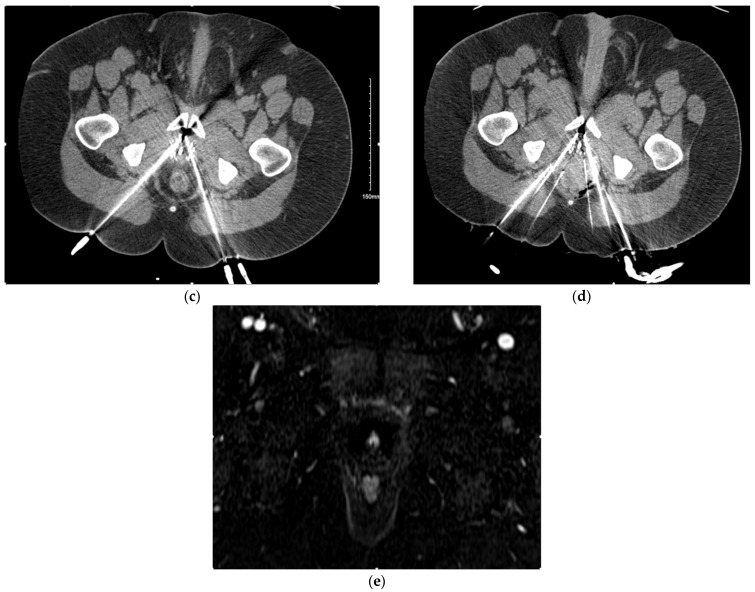
Case example of focal CT-guided cryoablation of locally recurrent prostate cancer. (**a**) Fused axial images of a fluciclovine-flourine-18-labeled SPECT/CT showing a focus of avidity along the midline anterior mid-gland, correlating with local prostate cancer recurrence. (**b**) Axial contrast-enhanced T1-weighted MRI subtraction image showing the enhancement of locally recurrent prostate cancer within the midline anterior mid-gland correlating with area of avidity (**a**). (**c**) Axial CT images showing multiple transgluteal IcePearl cryoablation needles within the target lesion within the midline anterior mid-gland. (**d**) Transgluteal cryoablation probe into the prostate target lesion after instillation of 2% contrast:0.9% saline solution within the perirectal region to safely separate critical structures for cryoablation after placement of multiple hydrodissection needles; images also demonstrate ice ball formation within the target lesion. (**e**) Axial contrast-enhanced T1-weighted MRI subtraction image showing resolution of enhancement along the targeted region post-ablation.

The majority of patients are positioned prone, and a combination of CT and ultrasound guidance are used for transgluteal and/or transperineal probe placement. Initial images are obtained with and without iodinated contrast before probe placement and multiplanar reformats are created. After identifying the target lesion, ablation is commenced by advancing cryoablation probes into the target lesion with CT fluoroscopic guidance as well as ultrasound guidance. Given the need to obtain an adequate margin at –40 degrees Celsius, as well as the long configuration of the prostate, multiple probes are necessary to cover the targeted region to a lethal temperature. Once the ablation probes are placed, hydrodissection is performed to create appropriate separation from the surrounding critical structures, including the rectum, ureter, bladder, and the neurovascular bundle, utilizing 17–18 G needles and the infusion of a solution containing iodinated contrast in normal saline (1:50 dilution) [55]. Repeat imaging is obtained as necessary to ensure that there is a safe window at all times during the ablation. After confirming appropriate needle placement and hydrodissection, cryoablation is initiated with intermittent imaging at two-minute intervals throughout the freeze cycle, and ablation is stopped when the lesion and margin are covered or if there is a concern for potential adjacent non-target thermal injury. Active thaw cycles are performed after every freeze cycle for a minimum of 10 min with intermittent imaging (Figure 1 and Figure 2). The authors’ institutional protocol is to perform at least 3 cycles of ablation for every patient when possible. After the final thaw cycle, all needles were removed, and repeat unenhanced CT imaging was obtained to confirm that there were no immediate complications.

The patient is then placed back in the supine position on the stretcher and the urethral warming catheter is left running for a minimum of 15 min prior to exchanging the catheter for an 18 F Foley catheter. Patients are discharged with the urinary catheter, which is removed five days after the procedure in the outpatient IR clinic. All patients are sent home with a standardized course of oral methylprednisolone and a five-day course of oral antibiotics (500 mg twice-daily oral ciprofloxacin).

While there are currently no standardized protocols for follow-up after ablation therapy, there are several initiatives to develop consensus guidelines [56]. In general, patients who have had primary treatment after focal therapy should resume close active surveillance but also obtain post-treatment biopsies and mpMRI imaging periodically for five years, as well as continued lifelong follow-up. In patients with recurrent disease, there are even fewer guideline-based recommendations, and our departmental guidelines recommend PSA, PSMA-PET, and mpMRI 3 months after initial treatment, 6 months after treatment, and annually thereafter.

### 3.3. Follow-Up

As focal therapy for prostate cancer has become more widespread in the primary treatment and salvage setting, there is an increasing need to standardize follow-up protocols. Unfortunately, the literature regarding follow-up strategies for focal therapy is scarce, and one of the primary limitations in many of the published trials in image-guided focal therapy for prostate cancer is the lack of a standardized follow-up protocol [57,58].

The author’s institution utilizes a follow-up protocol that incorporates expert consensus as well as institutional experience for a standardized follow-up protocol [58] Patients obtain a prostate-specific antigen (PSA) blood test in follow-up at 3 months post treatment, and subsequently every 3 months for the first year of treatment. After one year, the patient receives a PSA test every 6 months, as well as continued long-term follow-up with the treating physician and medical oncologist. Additionally, while the optimal role of mpMRI and PSMA-PET are unclear in the follow-up strategy for image-guided focal therapy, it is clear that there is a benefit to long-term follow-up with imaging. The author’s obtain an mpMRI and PSMA-PET at 3, 6, and 9 months post treatment then switch to imaging follow-up every 6 months for a total imaging follow-up time of 5 years. It is worth noting, that consensus opinion varies on this, with some suggesting that patients only need an MRI at 6 months and 12 months post focal therapy [58]. Our institution has adopted a more comprehensive protocol to make the focal therapy follow-up uniform for both primary and recurrent prostate cancer focal therapy patients. For patients being treated in the primary setting, an image-guided biopsy of the treated lesion to assess in-field recurrence/treatment failure and systematic biopsy of untreated regions to assess out of field recurrence should be performed at 6 and 12 months post-procedure [58]. For patients being treated in the salvage setting, a biopsy is not routinely performed at 6 and 12 months post focal-therapy; however, this is an on-going area of study. Finally, all patients have a standardized functional assessment after focal therapy to assess erectile function, continence, and urinary symptoms every 3 months after treatment until the patient either returns to their baseline functional status or has set a new baseline activity with stable symptoms. 

### 3.4. Limitations

The field of ablation and focal therapies of the prostate is rapidly evolving. This review provides a general overview of cryoablation, based on institutional expertise, as well as the fact that cryoablation is currently one of the most reported modalities in the urology and radiology literature using cross-sectional image guidance [32,43,44,46,47]. 

However, there are other imaging and thermal modalities that are currently available. For example, high intensity focused ultrasound (HIFU) demonstrates promising oncologic outcomes with a large study of 625 patients demonstrating a five-year metastasis-free survival of 98% [54]. A similar pooled analysis was performed demonstrating a 92% freedom from re-treatment rate at one year. However, there are potential concerns with HIFU due to the difficulty in the treatment of anterior gland lesions, concerns about erectile dysfunction, and fistula formation [59,60].

Focal laser ablation (FLA) of the prostate is a promising area of treatment that was evaluated in several small studies. One study of 120 patients for low- and intermediate-risk patients demonstrated an 83% freedom from retreatment rate at one year [61]. FLA also has an excellent safety profile; however, it is not widely available, is technically demanding, and has poor long-term follow-up [59,61,62]. 

Additional promising areas of investigation include irreversible electroporation (IRE) due to its exquisite ability to protect surrounding structures, however there is still a paucity of data in this space. Finally, the MRI Guided Transurethral Ultrasound Ablation (TULSA) platform is being actively evaluated with several studies recently reporting promising results because of the advantages of HIFU as well as MRI guidance and thermometry achieving intermediate term tumor control with minimal side effects [63,64]. 

## 4. Reported Outcomes

Much of the research to date has involved relatively small numbers of patients and retrospective analyses (Table 1). However, a recent prospective single-arm study by Fernández-Pascual et al. enrolled 75 patients with between one and three lesions targeted for treatment [42]. Treatment planning software was used, together with ultrasound and cryoablation, for the treatment of biopsy-confirmed prostate cancer. Of note, the authors defined recurrence as an infield lesion with clinically significant prostate cancer (Gleason ≥ 7). Using that definition, they reported that 56 of 66 patients had recurrence-free status. However, of the 50 patients who underwent follow-up biopsy, 23 (46%) had findings positive for prostate cancer. This highlights the challenges that exist with defining success for focal prostate therapy, which is arbitrary and varies between studies.

Another study by Baskin et al. retrospectively reviewed 75 patients who underwent prostate cryotherapy and went on to surveillance biopsy [65]. Thirty-eight of the seventy-five patients (50.7%) had a negative biopsy, but the remaining patients had biopsy specimens showing prostate cancer on the previously treated side (*n* = 13), untreated side (*n* = 17), or both sides (*n* = 7). The authors highlighted the fact that PSA and multiparametric MRI were not reflective of the presence or absence of residual disease on that follow-up biopsy.

In patients who have undergone prior cryoablation or external beam radiation, cryotherapy can also be used for salvage therapy [66]. Campbell et al. reviewed 126 patients, half of whom underwent primary external beam radiation and the other half underwent primary cryoablation. Both groups underwent salvage cryoablation for recurrence, with analysis showing no difference between the two groups for biochemical progression-free survival (BPFS) at 2- and 5-years.

Local salvage with cryotherapy can be performed in patients who have undergone prostatectomy or radiation as a primary treatment, with the potential for local control and low complication rates [67].

As with most invasive procedures, there are risks associated with prostate cryoablation. These include rare but potentially serious complications such as rectourethral fistula formation [44] and also others including urinary infection, transient dysuria, scrotal pain, urinary retention, and urinary incontinence [44,45]. In the study by De Marini et al., 60% of patients (*n* = 18) reported complications, of which 28% required surgical or other interventional treatments, and the remaining 72% were managed with conservative or pharmacological treatments [45]. It is important to note, however, that the complication rates reported are based on whole- or hemi-gland cryoablation rather than focal ablation. In recent reports of focal ablation, the complication rates are much lower; however, this warrants further study.

Other thermal and non-thermal modalities also offer potential in this group of patients. For example, a multicenter prospective trial looked at 37 patients with focal recurrent prostate cancer after external beam radiation or brachytherapy who were treated with irreversible electroporation (IRE) as salvage therapy [68] and found that 27 patients (73%) had no evidence of local or metastatic disease at the 12-month follow-up.

Finally, thermal ablation may play a role in patients with low- or intermediate-stage and grade prostate cancer. An increasing awareness of the potential for the overtreatment of lower-risk prostate cancer has led to several centers reporting more patients presenting with higher risk, more aggressive prostate cancer, a phenomenon known as stage migration [69,70,71]. The future research may provide additional insight into the potential role of thermal ablation in the management of patients with prostate cancer, specifically, which patients are most likely to benefit from ablative therapies.

## 5. Conclusions

In conclusion, image-guided focal treatment of prostate cancer is a potentially curative option for patients with primary prostate cancers and an additional treatment option for patients with recurrent disease. Although there are a wide variety of guidance and treatment modalities available, most radiologists are familiar with image-guided cryoablation, and the focus of this review should give interventional radiologists the necessary background to begin working in this space.

## Figures and Tables

**Table 1:** Selected publications outlining the use of thermal ablation for the management of prostate cancer.

Year	Authors	Whole Gland/Focal	Image Guidance	Thermal Modality	Number of Patients Treated	Type of Study	Reference
2002	Onik et al.	Focal	Ultrasound	Cryoablation	9	Retrospective	[8]
2012	Gangi et al.	Whole gland	MRI	Cryoablation	11	Retrospective	[44]
2012	Chopra et al.	Whole gland	MRI	HIFU	8	Retrospective	[64]
2012	Uddin Ahmed et al.	Whole gland	Ultrasound	HIFU	84	Retrospective	[31]
2013	Woodrum et al.	Focal	MRI	Cryoablation	18	Retrospective	[32]
2013	Bomers et al.	Focal	MRI	Cryoablation	10	Retrospective	[47]
2016	Eggener et al.	Focal	MRI	Laser	27	Phase 2 trial	[59]
2017	Klotz et al.	Whole gland	MRI	Laser	115	Prospective	[63]
2017	Valerio et al.	Focal	MRI/US fusion	Cryoablation	18	Prospective	[25]
2017	Overduin et al.	Focal	MRI	Cryoablation	47	Retrospective	[46]
2018	Kinsman et al.	Whole gland	MRI	Cryoablation	4	Retrospective	[43]
2019	De Marini et al.	Whole gland	MRI	Cryoablation	30	Retrospective	[45]
2019	Walser et al.	Focal	MRI	Laser	120	Prospective	[61]
2022	Ehdaie et al.	Focal	MRI	HIFU	101	Multicentre, phase 2b study	[10]
2022	Fernández-Pascual et al.	Focal	MRI/US fusion	Cryoablation	75	Prospective	[42]
2022	Baskin et al.	Focal	US	Cryoablation	75	Retrospective	[65]

Abbreviations: HIFU—high-intensity focused ultrasound.

## References

[1] Culp M.B., Soerjomataram I., Efstathiou J.A., Bray F., Jemal A. (2020). Recent Global Patterns in Prostate Cancer Incidence and Mortality Rates. Eur. Urol..

[2] Cooperberg M.R., Lubeck D.P., Meng M.V., Mehta S.S., Carroll P.R. (2004). The changing face of low-risk prostate cancer: Trends in clinical presentation and primary management. J. Clin. Oncol..

[3] Hamdy F.C., Donovan J.L., Lane J.A., Metcalfe C., Davis M., Turner E.L., Martin R.M., Young G.J., Walsh E.I., Bryant R.J. (2023). Fifteen-Year Outcomes after Monitoring, Surgery, or Radiotherapy for Prostate Cancer. N. Engl. J. Med..

[4] Lei J.H., Liu L.R., Wei Q., Yan S.B., Song T.R., Lin F.S., Yang L., Cao D.H., Yuan H.C., Xue W.B. (2015). Systematic review and meta-analysis of the survival outcomes of first-line treatment options in high-risk prostate cancer. Sci. Rep..

[5] Petrelli F., Vavassori I., Coinu A., Borgonovo K., Sarti E., Barni S. (2014). Radical prostatectomy or radiotherapy in high-risk prostate cancer: A systematic review and metaanalysis. Clin. Genitourin. Cancer.

[6] Hoffman K.E., Penson D.F., Zhao Z., Huang L.C., Conwill R., Laviana A.A., Joyce D.D., Luckenbaugh A.N., Goodman M., Hamilton A.S. (2020). Patient-Reported Outcomes through 5 Years for Active Surveillance, Surgery, Brachytherapy, or External Beam Radiation with or without Androgen Deprivation Therapy for Localized Prostate Cancer. JAMA.

[7] Mottet N., Bellmunt J., Bolla M., Briers E., Cumberbatch M.G., De Santis M., Fossati N., Gross T., Henry A.M., Joniau S. (2017). EAU-ESTRO-SIOG Guidelines on Prostate Cancer. Part 1: Screening, Diagnosis, and Local Treatment with Curative Intent. Eur. Urol..

[8] Onik G., Narayan P., Vaughan D., Dineen M., Brunelle R. (2002). Focal “nerve-sparing” cryosurgery for treatment of primary prostate cancer: A new approach to preserving potency. Urology.

[9] Onik G.M., Cohen J.K., Reyes G.D., Rubinsky B., Chang Z., Baust J. (1993). Transrectal ultrasound-guided percutaneous radical cryosurgical ablation of the prostate. Cancer.

[10] Ehdaie B., Tempany C.M., Holland F., Sjoberg D.D., Kibel A.S., Trinh Q.D., Durack J.C., Akin O., Vickers A.J., Scardino P.T. (2022). MRI-guided focused ultrasound focal therapy for patients with intermediate-risk prostate cancer: A phase 2b, multicentre study. Lancet Oncol..

[11] Woodrum D.A., Kawashima A., Gorny K.R., Mynderse L.A. (2019). Magnetic Resonance-Guided Prostate Ablation. Semin. Intervent. Radiol..

[12] Gardner T.A., Koch M.O. (2005). Prostate cancer therapy with high-intensity focused ultrasound. Clin. Genitourin. Cancer.

[13] Arcot R., Polascik T.J. (2022). Evolution of Focal Therapy in Prostate Cancer: Past, Present, and Future. Urol. Clin. N. Am..

[14] Bozzini G., Colin P., Nevoux P., Villers A., Mordon S., Betrouni N. (2013). Focal therapy of prostate cancer: Energies and procedures. Urol. Oncol..

[15] Valerio M., Cerantola Y., Eggener S.E., Lepor H., Polascik T.J., Villers A., Emberton M. (2017). New and Established Technology in Focal Ablation of the Prostate: A Systematic Review. Eur. Urol..

[16] Vickers A.J., Ulmert D., Sjoberg D.D., Bennette C.J., Björk T., Gerdtsson A., Manjer J., Nilsson P.M., Dahlin A., Bjartell A. (2013). Strategy for detection of prostate cancer based on relation between prostate specific antigen at age 40-55 and long term risk of metastasis: Case-control study. BMJ.

[17] Weinreb J.C., Barentsz J.O., Choyke P.L., Cornud F., Haider M.A., Macura K.J., Margolis D., Schnall M.D., Shtern F., Tempany C.M. (2016). PI-RADS Prostate Imaging—Reporting and Data System: 2015, Version 2. Eur. Urol..

[18] Hambrock T., Somford D.M., Huisman H.J., van Oort I.M., Witjes J.A., Hulsbergen-van de Kaa C.A., Scheenen T., Barentsz J.O. (2011). Relationship between apparent diffusion coefficients at 3.0-T MR imaging and Gleason grade in peripheral zone prostate cancer. Radiology.

[19] Rouvière O., Puech P., Renard-Penna R., Claudon M., Roy C., Mège-Lechevallier F., Decaussin-Petrucci M., Dubreuil-Chambardel M., Magaud L., Remontet L. (2019). Use of prostate systematic and targeted biopsy on the basis of multiparametric MRI in biopsy-naive patients (MRI-FIRST): A prospective, multicentre, paired diagnostic study. Lancet Oncol..

[20] Kasivisvanathan V., Rannikko A.S., Borghi M., Panebianco V., Mynderse L.A., Vaarala M.H., Briganti A., Budäus L., Hellawell G., Hindley R.G. (2018). MRI-Targeted or Standard Biopsy for Prostate-Cancer Diagnosis. N. Engl. J. Med..

[21] Heidenreich A., Bastian P.J., Bellmunt J., Bolla M., Joniau S., van der Kwast T., Mason M., Matveev V., Wiegel T., Zattoni F. (2014). EAU guidelines on prostate cancer. part 1: Screening, diagnosis, and local treatment with curative intent-update 2013. Eur. Urol..

[22] Mottet N., van den Bergh R.C.N., Briers E., Van den Broeck T., Cumberbatch M.G., De Santis M., Fanti S., Fossati N., Gandaglia G., Gillessen S. (2021). EAU-EANM-ESTRO-ESUR-SIOG Guidelines on Prostate Cancer-2020 Update. Part 1: Screening, Diagnosis, and Local Treatment with Curative Intent. Eur. Urol..

[23] Wang A., O’Connor L.P., Yerram N.K., Nandanan N., Ahdoot M., Lebastchi A.H., Gurram S., Chalfin H., Pinto P.A. (2020). Focal therapy for prostate cancer: Recent advances and future directions. Clin. Adv. Hematol. Oncol..

[24] Martin R.M., Donovan J.L., Turner E.L., Metcalfe C., Young G.J., Walsh E.I., Lane J.A., Noble S., Oliver S.E., Evans S. (2018). Effect of a Low-Intensity PSA-Based Screening Intervention on Prostate Cancer Mortality: The CAP Randomized Clinical Trial. JAMA.

[25] Valerio M., Shah T.T., Shah P., Mccartan N., Emberton M., Arya M., Ahmed H.U. (2017). Magnetic resonance imaging-transrectal ultrasound fusion focal cryotherapy of the prostate: A prospective development study. Urol. Oncol..

[26] Wallis C.J.D., Saskin R., Choo R., Herschorn S., Kodama R.T., Satkunasivam R., Shah P.S., Danjoux C., Nam R.K. (2016). Surgery Versus Radiotherapy for Clinically-localized Prostate Cancer: A Systematic Review and Meta-analysis. Eur. Urol..

[27] Potosky A.L., Davis W.W., Hoffman R.M., Stanford J.L., Stephenson R.A., Penson D.F., Harlan L.C. (2004). Five-year outcomes after prostatectomy or radiotherapy for prostate cancer: The prostate cancer outcomes study. J. Natl. Cancer Inst..

[28] Freedland S.J., Humphreys E.B., Mangold L.A., Eisenberger M., Dorey F.J., Walsh P.C., Partin A.W. (2005). Risk of prostate cancer-specific mortality following biochemical recurrence after radical prostatectomy. JAMA.

[29] Trock B.J., Han M., Freedland S.J., Humphreys E.B., DeWeese T.L., Partin A.W., Walsh P.C. (2008). Prostate cancer-specific survival following salvage radiotherapy vs observation in men with biochemical recurrence after radical prostatectomy. JAMA.

[30] Stephenson A.J., Scardino P.T., Kattan M.W., Pisansky T.M., Slawin K.M., Klein E.A., Anscher M.S., Michalski J.M., Sandler H.M., Lin D.W. (2007). Predicting the outcome of salvage radiation therapy for recurrent prostate cancer after radical prostatectomy. J. Clin. Oncol..

[31] Uddin Ahmed H., Cathcart P., Chalasani V., Williams A., McCartan N., Freeman A., Kirkham A., Allen C., Chin J., Emberton M. (2012). Whole-gland salvage high-intensity focused ultrasound therapy for localized prostate cancer recurrence after external beam radiation therapy. Cancer.

[32] Woodrum D.A., Kawashima A., Karnes R.J., Davis B.J., Frank I., Engen D.E., Gorny K.R., Felmlee J.P., Callstrom M.R., Mynderse L.A. (2013). Magnetic resonance imaging-guided cryoablation of recurrent prostate cancer after radical prostatectomy: Initial single institution experience. Urology.

[33] Gotto G.T., Yunis L.H., Vora K., Eastham J.A., Scardino P.T., Rabbani F. (2010). Impact of prior prostate radiation on complications after radical prostatectomy. J. Urol..

[34] Heidenreich A., Bastian P.J., Bellmunt J., Bolla M., Joniau S., van der Kwast T., Mason M., Matveev V., Wiegel T., Zattoni F. (2014). EAU guidelines on prostate cancer. Part II: Treatment of advanced, relapsing, and castration-resistant prostate cancer. Eur. Urol..

[35] Duchesne G.M., Woo H.H., Bassett J.K., Bowe S.J., D’Este C., Frydenberg M., King M., Ledwich L., Loblaw A., Malone S. (2016). Timing of androgen-deprivation therapy in patients with prostate cancer with a rising PSA (TROG 03.06 and VCOG PR 01-03 [TOAD]): A randomised, multicentre, non-blinded, phase 3 trial. Lancet Oncol..

[36] Keating N.L., O’Malley A.J., Freedland S.J., Smith M.R. (2010). Diabetes and cardiovascular disease during androgen deprivation therapy: Observational study of veterans with prostate cancer. J. Natl. Cancer Inst..

[37] Saigal C.S., Gore J.L., Krupski T.L., Hanley J., Schonlau M., Litwin M.S., Project U.D.i.A. (2007). Androgen deprivation therapy increases cardiovascular morbidity in men with prostate cancer. Cancer.

[38] Crawford E.D., Moul J.W. (2015). ADT risks and side effects in advanced prostate cancer: Cardiovascular and acute renal injury. Oncology.

[39] Nanda A., Chen M.H., Braccioforte M.H., Moran B.J., D’Amico A.V. (2009). Hormonal therapy use for prostate cancer and mortality in men with coronary artery disease-induced congestive heart failure or myocardial infarction. JAMA.

[40] Ahmed H.U. (2009). The index lesion and the origin of prostate cancer. N. Engl. J. Med..

[41] Algaba F., Montironi R. (2010). Impact of prostate cancer multifocality on its biology and treatment. J. Endourol..

[42] Fernández-Pascual E., Manfredi C., Martín C., Martínez-Ballesteros C., Balmori C., Lledó-García E., Quintana L.M., Curvo R., Carballido-Rodríguez J., Bianco F.J. (2022). mpMRI-US Fusion-Guided Targeted Cryotherapy in Patients with Primary Localized Prostate Cancer: A Prospective Analysis of Oncological and Functional Outcomes. Cancers.

[43] Kinsman K.A., White M.L., Mynderse L.A., Kawashima A., Rampton K., Gorny K.R., Atwell T.D., Felmlee J.P., Callstrom M.R., Woodrum D.A. (2018). Whole-Gland Prostate Cancer Cryoablation with Magnetic Resonance Imaging Guidance: One-Year Follow-Up. Cardiovasc. Intervent. Radiol..

[44] Gangi A., Tsoumakidou G., Abdelli O., Buy X., de Mathelin M., Jacqmin D., Lang H. (2012). Percutaneous MR-guided cryoablation of prostate cancer: Initial experience. Eur. Radiol..

[45] De Marini P., Cazzato R.L., Garnon J., Tricard T., Koch G., Tsoumakidou G., Ramamurthy N., Lang H., Gangi A. (2019). Percutaneous MR-guided whole-gland prostate cancer cryoablation: Safety considerations and oncologic results in 30 consecutive patients. Br. J. Radiol..

[46] Overduin C.G., Jenniskens S.F.M., Sedelaar J.P.M., Bomers J.G.R., Fütterer J.J. (2017). Percutaneous MR-guided focal cryoablation for recurrent prostate cancer following radiation therapy: Retrospective analysis of iceball margins and outcomes. Eur. Radiol..

[47] Bomers J.G., Yakar D., Overduin C.G., Sedelaar J.P., Vergunst H., Barentsz J.O., de Lange F., Fütterer J.J. (2013). MR imaging-guided focal cryoablation in patients with recurrent prostate cancer. Radiology.

[48] Eastham J.A., Auffenberg G.B., Barocas D.A., Chou R., Crispino T., Davis J.W., Eggener S., Horwitz E.M., Kane C.J., Kirkby E. (2022). Clinically Localized Prostate Cancer: AUA/ASTRO Guideline, Part I: Introduction, Risk Assessment, Staging, and Risk-Based Management. J. Urol..

[49] Ginsburg K.B., Elshafei A., Yu C., Jones J.S., Cher M.L. (2017). Avoidance of androgen deprivation therapy in radiorecurrent prostate cancer as a clinically meaningful endpoint for salvage cryoablation. Prostate.

[50] de la Rosette J., Ahmed H., Barentsz J., Johansen T.B., Brausi M., Emberton M., Frauscher F., Greene D., Harisinghani M., Haustermans K. (2010). Focal therapy in prostate cancer-report from a consensus panel. J. Endourol..

[51] Edison E., Tariq Shah T., Ahmed H.U. (2017). Focal Ablation of Early-Stage Prostate Cancer: Candidate Selection, Treatment Guidance, and Assessment of Outcome. Urol. Clin. N. Am..

[52] Tay K.J., Polascik T.J., Elshafei A., Tsivian E., Jones J.S. (2017). Propensity Score-Matched Comparison of Partial to Whole-Gland Cryotherapy for Intermediate-Risk Prostate Cancer: An Analysis of the Cryo On-Line Data Registry Data. J. Endourol..

[53] Uchida T., Shoji S., Nakano M., Hongo S., Nitta M., Usui Y., Nagata Y. (2011). High-intensity focused ultrasound as salvage therapy for patients with recurrent prostate cancer after external beam radiation, brachytherapy or proton therapy. BJU Int..

[54] Phillips R., Shi W.Y., Deek M., Radwan N., Lim S.J., Antonarakis E.S., Rowe S.P., Ross A.E., Gorin M.A., Deville C. (2020). Outcomes of Observation vs Stereotactic Ablative Radiation for Oligometastatic Prostate Cancer: The ORIOLE Phase 2 Randomized Clinical Trial. JAMA Oncol..

[55] Campbell C., Lubner M.G., Hinshaw J.L., Muñoz del Rio A., Brace C.L. (2012). Contrast media-doped hydrodissection during thermal ablation: Optimizing contrast media concentration for improved visibility on CT images. AJR Am. J. Roentgenol..

[56] Tay K.J., Amin M.B., Ghai S., Jimenez R.E., Kench J.G., Klotz L., Montironi R., Muto S., Rastinehad A.R., Turkbey B. (2019). Surveillance after prostate focal therapy. World J. Urol..

[57] Muller B.G., van den Bos W., Brausi M., Fütterer J.J., Ghai S., Pinto P.A., Popeneciu I.V., de Reijke T.M., Robertson C., de la Rosette J.J. (2015). Follow-up modalities in focal therapy for prostate cancer: Results from a Delphi consensus project. World J. Urol..

[58] Lebastchi A.H., George A.K., Polascik T.J., Coleman J., de la Rosette J., Turkbey B., Wood B.J., Gorin M.A., Sidana A., Ghai S. (2020). Standardized Nomenclature and Surveillance Methodologies After Focal Therapy and Partial Gland Ablation for Localized Prostate Cancer: An International Multidisciplinary Consensus. Eur. Urol..

[59] Eggener S.E., Yousuf A., Watson S., Wang S., Oto A. (2016). Phase II Evaluation of Magnetic Resonance Imaging Guided Focal Laser Ablation of Prostate Cancer. J. Urol..

[60] Guillaumier S., Peters M., Arya M., Afzal N., Charman S., Dudderidge T., Hosking-Jervis F., Hindley R.G., Lewi H., McCartan N. (2018). A Multicentre Study of 5-year Outcomes Following Focal Therapy in Treating Clinically Significant Nonmetastatic Prostate Cancer. Eur. Urol..

[61] Walser E., Nance A., Ynalvez L., Yong S., Aoughsten J.S., Eyzaguirre E.J., Williams S.B. (2019). Focal Laser Ablation of Prostate Cancer: Results in 120 Patients with Low- to Intermediate-Risk Disease. J. Vasc. Interv. Radiol..

[62] Ahdoot M., Lebastchi A.H., Turkbey B., Wood B., Pinto P.A. (2019). Contemporary treatments in prostate cancer focal therapy. Curr. Opin. Oncol..

[63] Klotz L., Pavlovich C.P., Chin J., Hatiboglu G., Koch M., Penson D., Raman S., Oto A., Fütterer J., Serrallach M. (2021). Magnetic Resonance Imaging-Guided Transurethral Ultrasound Ablation of Prostate Cancer. J. Urol..

[64] Chopra R., Colquhoun A., Burtnyk M., N’djin W.A., Kobelevskiy I., Boyes A., Siddiqui K., Foster H., Sugar L., Haider M.A. (2012). MR imaging-controlled transurethral ultrasound therapy for conformal treatment of prostate tissue: Initial feasibility in humans. Radiology.

[65] Baskin A., Charondo L.B., Balakrishnan A., Cowan J.E., Cooperberg M.R., Carroll P.R., Nguyen H., Shinohara K. (2022). Medium Term Outcomes of Focal Cryoablation for Intermediate and High Risk Prostate Cancer: MRI and PSA are Not Predictive of Residual or Recurrent Disease. Urol. Oncol..

[66] Campbell S.P., Deivasigamani S., Arcot R., Adams E.S., Orabi H., Elshafei A., Tan W.P., Davis L., Wu Y., Chang A. (2023). Salvage Cryoablation for Recurrent Prostate Cancer Following Primary External Beam Radiotherapy or Primary Cryotherapy: A Propensity Score Matched Analysis of Mid-term Oncologic and Functional Outcomes. Clin. Genitourin. Cancer.

[67] Cornelis F., Havez M., Le Bras Y., Descat E., Richaud P., Grenier N. (2013). Salvage CT-guided transgluteal cryoablation for locally recurrent prostate cancer: Initial experiences. J. Vasc. Interv. Radiol..

[68] Blazevski A., Geboers B., Scheltema M.J., Gondoputro W., Doan P., Katelaris A., Agrawal S., Baretto D., Matthews J., Haynes A.M. (2023). Salvage irreversible electroporation for radio-recurrent prostate cancer—The prospective FIRE trial. BJU Int..

[69] Haas J.A., Mendez C., Sanchez A., Mirza A., Carpenter T.J., Witten M.R., Demircioglu G., Katz A.E., Repka M.C., Blacksburg S.R. (2019). Evidence of Stage Migration to Higher Risk Prostate Cancer and its Financial Implications in a Single Institution. Int. J. Radiat. Oncol. Biol. Phys..

[70] Boehm K., Borgmann H., Ebert T., Höfner T., Khaljani E., Schmid M., Schulze-Seemann W., Weib P., Herden J. (2021). Stage and Grade Migration in Prostate Cancer Treated with Radical Prostatectomy in a Large German Multicenter Cohort. Clin. Genitourin. Cancer.

[71] Di Mauro E., Di Bello F., Califano G., Morra S., Creta M., Celentano G., Abate M., Fraia A., Pezone G., Marino C. (2023). Incidence and Predicting Factors of Histopathological Features at Robot-Assisted Radical Prostatectomy in the mpMRI Era: Results of a Single Tertiary Referral Center. Medicina.

